# Automatic Detection and Classification of Natural Weld Defects Using Alternating Magneto-Optical Imaging and ResNet50

**DOI:** 10.3390/s24237649

**Published:** 2024-11-29

**Authors:** Yanfeng Li, Pengyu Gao, Yongbiao Luo, Xianghan Luo, Chunmei Xu, Jiecheng Chen, Yanxi Zhang, Genxiang Lin, Wei Xu

**Affiliations:** 1 School of Automobile and Transportation Engineering, Guangdong Polytechnic Normal University, Guangzhou 510632, China; 15815380750@163.com (Y.L.); x2234140821@163.com (X.L.); 25996@random.com (J.C.); lingengxiang@gpnu.edu.cn (G.L.); xuy_wei@126.com (W.X.); 2 Guangdong Provincial Welding Engineering Technology Research Center, Guangdong University of Technology, Guangzhou 510006, China; perrygao99@gmail.com (P.G.); yanxizhang@126.com (Y.Z.); 3 School of Computer Science and Technology, Southwest University of Science and Technology, Mianyang 621010, China; xchm@swust.edu.cn

**Keywords:** magneto-optical imaging, natural weld defect, alternating magnetic field, convolutional neural network, ResNet50

## Abstract

It is difficult to detect and identify natural defects in welded components. To solve this problem, according to the Faraday magneto-optical (MO) effect, a nondestructive testing system for MO imaging, excited by an alternating magnetic field, is established. For the acquired MO images of crack, pit, lack of penetration, gas pore, and no defect, Gaussian filtering, bilateral filtering, and median filtering are applied for image preprocessing. The effectiveness of these filtering methods is evaluated using metrics such as peak signal–noise ratio (PSNR) and mean squared error. Principal component analysis (PCA) is employed to extract column vector features from the downsampled defect MO images, which then serve as the input layer for the error backpropagation (BP) neural network model and the support vector machine (SVM) model. These two models can be used for the classification of partial defect MO images, but the recognition accuracy for cracks and gas pores is comparatively low. To further enhance the classification accuracy of natural weld defects, a convolutional neural network (CNN) classification model and a ResNet50 classification model for MO images of natural weld defects are established, and the model parameters are evaluated and optimized. The experimental results show that the overall classification accuracy of the ResNet50 model is 99%. Compared with the PCA-SVM model and CNN model, the overall classification accuracy was increased by 7.4% and 1.8%, and the classification accuracy of gas pore increased by 10% and 4%, respectively, indicating that the ResNet50 model can effectively and accurately classify natural weld defects.

## 1. Introduction

Welding technologies play a crucial role in modern manufacturing, especially in high-precision assembly and electronic component fabrication [1,2,3,4]. With advancements in welding processes, the demand for high-quality welds has increased, making defect detection particularly critical. Traditional methods such as ultrasonic testing and radiographic testing are already mature but have limitations, including an insufficient ability to identify complex defects and radiation risks during the detection process [5,6]. Magneto-optical (MO) imaging technology, with its advantages of no radiation and high sensitivity to minor defects, is increasingly becoming a key technique in weld defect detection [7,8]. This technology has significant advantages in detecting subsurface defects, with a detection depth of up to 6 mm, and its experimental costs are low [9]. The conventional MO detection method uses a direct current (DC) magnetic field to excite the weldments; because the magnetic field strength and direction of the direct current magnetic field are constant, some useful weld information may be lost, and it is easy to saturate [10]. The acquisition method of MO images of weld defects under alternating magnetic field excitation can collect dynamic MO images, thereby improving the problem of magnetic field information loss. Independent component analysis is used to achieve denoising of MO images and compare the denoising performance with dynamic filtering [11]. A dynamic MO imaging system is used to detect weld defects, and an MO image detection model for weld defects is established through support vector machine (SVM) models. The defect classification accuracy is only 91.5%, with which, it is difficult to meet the detection requirements [12]. The processing and analysis of MO imaging data remains challenging, especially in efficiently and accurately extracting and classifying defect features from complex images [13]. To address these issues, researchers are exploring solutions that combine advanced deep learning techniques to improve the effectiveness of MO imaging in defect detection.

The core of deep learning lies in neural network models, which require the construction of various neural network models to solve different problems [14,15]. Through self-learning, it can obtain highly abstract feature information that cannot be extracted manually. All neural network models will encounter the problem of generalization. The problem of generalization mainly focuses on enriching the sample space, limiting the hypothesis space, and changing the optimization objective. To enrich the sample space is to expand the sample space and increase the inductive bias of the model. Limiting the hypothesis space refers to reducing the risk of model overfitting by restricting the parameters and structure of the model. Changing the optimization objective means changing the traditional method to minimize the empirical risk on the training set as the optimization objective. By enhancing data through methods such as rotation, cropping, flipping, and brightness adjustment, the small sample problem is solved through data augmentation, so that the generalization ability of the weld image analysis model is improved [16,17]. An artificial neural network (ANN) with modified performance function is studied for automatic recognition and detection of weld defects in radiographic images [18,19]. A defect classification method based on direct multi-class support vector machine is proposed, which aims to minimize structural risk as the optimization objective and improve the generalization ability of the weld seam radiographic image analysis model in the case of a small training set [20,21]. Transfer learning in deep learning models can avoid overfitting, solve the problem of using small datasets, and extract the features of the images to be classified [22]. Several pre-trained networks, such as VGG16, ResNet50, DenseNet [23], AlexNet [24], and others, have served as feature extractors in classification problems [25]. The introduction of VGG-Net [26] and ResNet [27] further promotes the performance improvement of classification networks. As the depth of the network increases, ResNet improves the problem of gradient vanishing during backpropagation. A new CNN model based on ResNet50 is proposed to classify the defects in the radiographic images. Techniques such as stratified cross-validation, data augmentation, and regularization will be chosen to improve the model generalization ability and avoid overfitting [28]. A TOFD image weld defect detection method based on multi-image fusion and feature hybrid enhancement is proposed by combining deep learning techniques with domain knowledge in the field of TOFD detection [29]. A defect classification model for SMT welding images based on an improved ResNet model, namely the ResNet-34-ECA model, was proposed, with an overall classification accuracy of 98.2% [30]. In the field of aircraft engines, deep learning algorithms are also widely used. A hierarchical health monitoring model called the adaptive thresholding and coordinate-attention-based tree-inspired network (ATCATN) has been developed for the health monitoring of aero-engine bearings under strong background noise. The experimental results show that this model can accurately identify the fault locations and sizes of aero-engine bearings even under strong noise interference [31]. A data-driven time–frequency analysis (TFA) technology for CTNet was developed, which combines a fully convolutional auto-encoder network with the convolutional block attention module (CBAM). The experimental results show that the CTNet has good ability to detect wind turbine faults [32]. Deep learning algorithms can automatically learn and extract the features in images to effectively complete the tasks such as image classification and object detection [33].

At present, there is increasing international attention on the application of MO imaging technology. One study proposed using an improved remanence/magneto-optical imaging combined with a cost-sensitive convolutional neural network (CNN) for automatic defect detection and classification of low-carbon steel WAAM components [34]. The study demonstrated that this method significantly improved detection accuracy for surface defects in low-carbon steel products. Another study developed an MO imaging nondestructive testing system based on alternating magnetic field excitation, which effectively addressed the issue of detecting hidden weld defects. By establishing a three-dimensional finite element model and extracting texture features using gray-level co-occurrence matrix (GLCM) and Tamura methods, the researchers successfully applied a backpropagation (BP) neural network for defect classification, achieving an overall classification accuracy of 91.1% [35]. Additionally, research has focused on image fusion technology, using pixel standard-deviation-based image fusion methods to enhance the visual effect and detection accuracy of MO imaging. This method significantly improved image quality and showed promising results in weld defect detection [36].

There are still relatively few MO imaging weld defect detection methods based on deep learning, and they do not take into account the fact that images need to be “dynamically” observed during the evaluation process, resulting in low accuracy and credibility in the defect detection results. This paper aims to explore automatic detection and classification methods for natural weld defects by combining alternating MO imaging technology with neural network algorithms. Classification models including BP neural networks, SVM model, CNN, and ResNet50 are utilized for the recognition and classification of natural weld defects. An alternating magnetic field excitation imaging system based on Faraday MO effect is constructed to capture MO images of various weld defects. The collected MO images were preprocessed using a Gaussian filter, a bilateral filter, and a median filter, and the effectiveness of the different filtering techniques was evaluated. Image features were extracted using principal component analysis (PCA), and these features were input into the BP neural network and the SVM model for classification. These two models can be used for the classification of partial defect MO images, but the recognition accuracy of cracks and pores is relatively low. To improve the classification accuracy, the CNN-based model and the ResNet50 model were established, and the model parameters were optimized. The experimental results show that the overall classification accuracy of the CNN and ResNet50 models is 97.2% and 99%, respectively. Compared with different detection methods, the deep learning methods used for natural welded defects detection and recognition demonstrates stronger noise resilience, and can maintain high detection and recognition accuracy even under the significant deformation of welded defects.

The paper is organized as follows. Section 2 introduces the weld defect test system based on MO imaging and analyzes the mechanism of MO imaging. Section 3 presents the PCA method to extract the principal components of the column vector, establishes the PCA-BP classification model and the PCA-SVM classification model, and compares the classification performance of the models. Section 4 studies the CNN classification model and the ResNet50 classification model, explores the parameter settings of the two models, and compares and analyzes the classification performance of the models. Section 5 draws conclusions.

## 2. Experimental Methods

### 2.1. Experimental Setup

**Figure 1:**
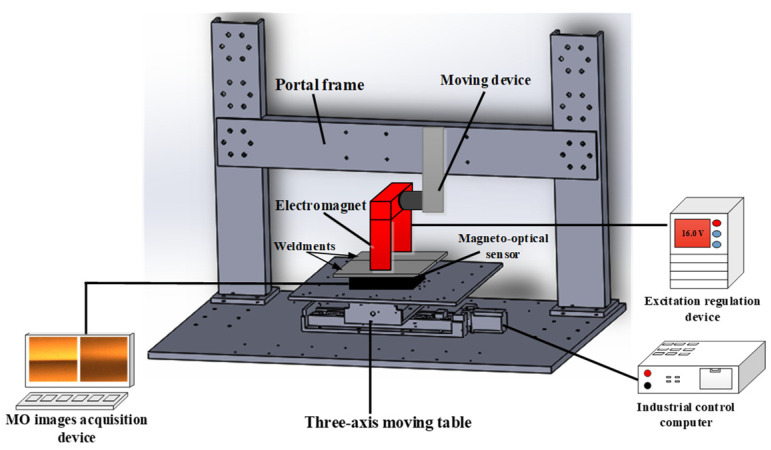
Schematic diagram of MO imaging detection system for welded defects excited by alternating magnetic field.

**Figure 2:**
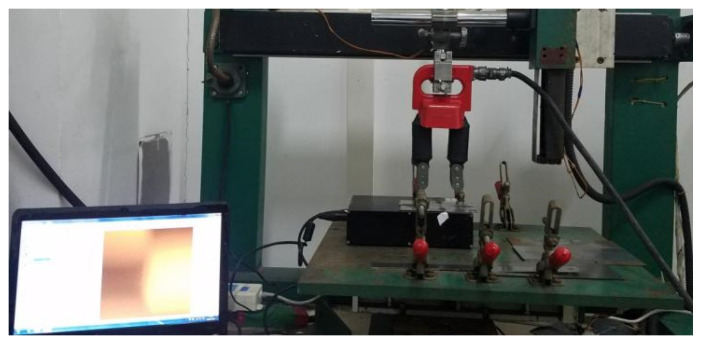
Physical diagram of MO imaging nondestructive testing platform for weld defects excited by alternating magnetic field.

The MO imaging detection device based on the leakage magnetic characteristics of natural weld defects primarily consists of the tested weldment, an excitation mechanism, an MO imaging sensor, a testing platform, and computer storage equipment. The schematic diagram of the MO imaging detection system for welded defects excited by alternating magnetic field is shown in Figure 1. The welding materials were carbon steel plate (No. 45) and high-strength steel (HSS). The excitation mechanism is an alternating current (AC) excitation system that generates an alternating magnetic field by connecting AC power to the excitation coil. The magnitude and direction of the alternating magnetic field will change periodically with the change in time. The MO testing platform uses a three-axis linkage motion platform, which can achieve motion and detection of the three directions of the X, Y, and Z axes, as shown in Figure 2. The MO sensor is mainly composed of a light-emitting diode (LED) light, an optical conduction system, a CMOS, and an MO film, with a size of 20 × 15 mm^2^, and its surface is coated with a mirror layer. The primary parameters of the MO sensor are detailed in Table 1.

The weldment material was selected from the HSS plate with a length of 150 mm, a width of 50 mm, and a thickness of 12 mm. Lack of penetration and pit were formed by the YAG laser welding machine on the abutting steel plate. The laser power is 10 kW. The defocus amount is −1 mm and the welding speed is 3 m/s. The shielding gas is argon, and the gas flow rate is 30 L/min. The angle between the gas nozzle and the torch is 45°. Under high-power laser welding conditions, there is a deviation of 2 mm and −2 mm between the starting and ending positions of the laser beam and the weld seam during welding, resulting in pits and incomplete penetration of the test weldment. The size and shape of surface pits on welded parts can be measured, but the depth and location of incomplete penetration cannot be predicted. Natural welding defects were formed on a carbon steel plate (No. 45) by TIG welding. The welding current of the TIG machine was adjusted within the range of 80 A–130 A, and the welding speeds for the initial welding tests were set to 1, 1.5, 2, and 2.5 mm/s. At the same time, different types of natural weld defects were obtained by rapidly cooling the high-temperature-welded specimens that had completed the welding operation, including crack, pit, and incomplete penetration.

### 2.2. Principle of MO Imaging for Weld Defects

**Figure 3:**
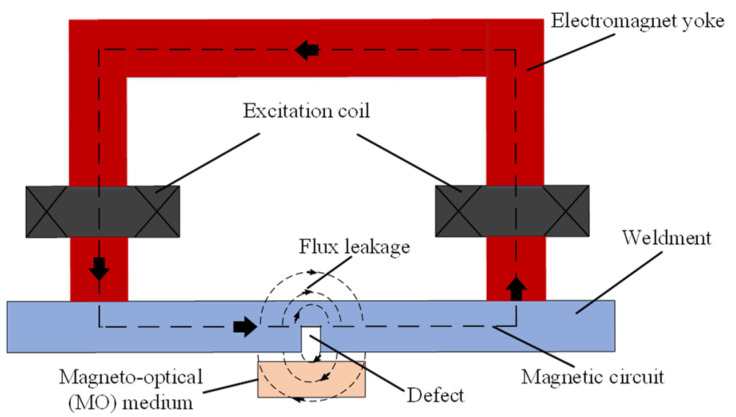
Schematic diagram of leakage magnetic field at welded defect.

**Figure 4:**
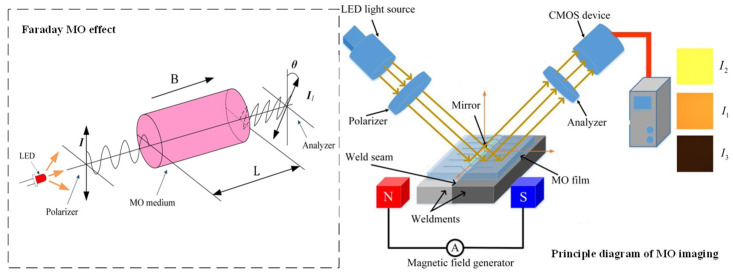
Principle diagram of MO imaging detection for weld defects.

The principle of MO imaging for natural weld defects mainly involves the concepts of leakage magnetic fields [37], the Faraday MO effect [38], and the application of external excitation magnetic fields, as illustrated in Figure 3 and Figure 4. When an external magnetic field is applied to a ferromagnetic weldment with high magnetic permeability, it becomes magnetized. If there are defects on the surface or inside of the weldment, the local magnetic permeability of the weldment material will decrease, and the magnetic resistance will increase, resulting in a non-uniform magnetic flux distribution. The magnetic field lines that pass through the weld defect are distorted, with a portion of the magnetic field lines passing through the weldment and another portion overflowing into the air and returning to the weldment, forming a leakage magnetic field in the local area of the weld defect’s surface, as shown in Figure 3. As the shape and size of the weld defect change, the magnitude of the leakage magnetic field varies accordingly. MO imaging equipment is used to detect changes in the leakage magnetic field and convert these signals into corresponding MO images. By applying image processing algorithms to extract defect information from MO images, the category of defects can be determined.

Faraday MO rotation effect means that when a beam of linearly polarized light travels through an MO medium. If an external magnetic field is applied along the direction of light propagation, the vibration surface of the polarized light rotates by a Faraday rotation angle ***θ***. Although the trajectory of linearly polarized light does not change in a strong magnetic field, it will deflect in the presence of a leakage magnetic field generated by defects. A polarizer detects this deflection angle, and a CMOS camera captures the light intensity. The left side of Figure 4 is a schematic diagram of Faraday MO rotation effect, where the Faraday rotation angle ***θ*** is proportional to the magnitude of the external magnetic field, the effective path of the light through the MO medium and the material properties of the MO medium. The rotation direction of the incident polarized light is solely dependent on the direction of the external magnetic field. The magnitude of ***θ*** can be expressed as follows:

***θ*** = *V**B**L*_m_ (1)

where ***B*** is the magnetic flux density and *L*_m_ is the effective path length of the polarized light through the MO medium. *V* is the Verdet constant of the MO medium.

The vertical component of the leakage magnetic field ***B***_z_ perpendicular to the weldment is the primary cause of deflection. The weldment is located in the external magnetic field generated by the magnetic field generator. In the defect-free area, the weldment will form a complete magnetic circuit, and there will be no vertical magnetic field along the propagation direction of polarized light. The vibration surface of polarized light will not deflect. When there are defects, the weldment cannot form a complete magnetic circuit, and the vertical magnetic field component at the defect below the reflective mirror surface changes. If there is a vertical magnetic field along the propagation direction of the polarized light, the vibration surface of the polarized light will be deflected. The linearly polarized light containing welding quality information is received by the CMOS sensor after passing through a polarizer, forming an MO image of the weld defect, as shown on the right side of Figure 4. When the direction of the external magnetic field is toward the north pole, the plane of polarization rotates clockwise by a Faraday rotation angle along the direction of light propagation. When the direction of the magnetic field is toward the south pole, the plane of polarization rotates counterclockwise by a Faraday rotation angle ***θ***.

By applying AC power to the magnetic field generator can generate an alternating magnetic field at both ends of the magnetic yoke that changes in direction and magnitude at a certain frequency, and the Faraday rotation angle also changes accordingly. The light intensity detected by the analyzer is represented as follows:

(2) $$I_{1}=I_{0}\cos^{2}\alpha$$

(3) $$I_{2}=I_{0}\cos^{2}(\alpha -\theta )$$

(4) $$I_{3}=I_{0}\cos^{2}(\alpha +\theta )$$

where *I*_0_ is the intensity of the incident linearly polarized light, and *α* is the inherent rotation degree of the linearly polarized light without the application of an external magnetic field. These equations show how the intensity of light detected by the polarizer varies with the direction and intensity of the magnetic field, reflecting the corresponding variations in the Faraday rotation angle [39].

### 2.3. Preprocessing of MO Images for Weld Defect Analysis

Each image filtering algorithm has its applicability. It is extremely difficult to judge the filtering effect of MO images by observing the visual effect of the filtered image through human visual perception, and this method is time-consuming and labor-intensive [40]. Therefore, by comparing the mean square error (MSE), the peak signal–noise ratio (PSNR), the structural similarity (SSIM), and the filtering time between the filtered image and the original image, the quality of the image filtering effect can be determined. This section studied Gaussian filtering, bilateral filtering, and median filtering algorithms, and used these three algorithms to filter MO images. The filtering effect was evaluated by MSE, PSNR, and SSIM.

MSE is an indicator used to evaluate the difference between the original image and the filtered image. MSE calculates the average of the squared differences in pixel values between the original image and the filtered image, as follows:

(5) $$\mathrm{MSE}=\frac{1}{mn}\Sigma_{i=0}^{m-1}\Sigma_{j=0}^{n-1}{\left(I_{\left(i,j\right)}-K_{\left(i,j\right)}\right)}^{2}$$

where m and n denote the number of rows and columns of the image, respectively, $I_{\left(i,j\right)}\;$ is the pixel value of the original image at (i,j), and $K_{\left(i,j\right)}$ is the pixel value of the filtered image at (i,j). Therefore, a smaller MSE indicates that the filtered image approximates the original image more closely, reflecting a higher image quality.

PSNR is the ratio between the maximum possible value (peak signal) of an image and the distortion of the image, and the formula is:

(6) $$\mathrm{PSNR}=10\log_{10}\left(\frac{MAX^{2}}{MSE}\right)$$

where MAX represents the maximum value of the image pixels, typically 255. The unit of PSNR is decibels (dB), and the higher the value, the smaller the distortion and the higher the image quality.

SSIM can be used to compare the structural similarity between two images, and its formula is:

(7) $$\mathrm{SSIM}\left(x,y\right)=\frac{\left(2u_{x}u_{y}+C_{1}\right)\left(2\sigma_{xy}+C_{2}\right)}{\left(\mu_{x}^{2}+\mu_{y}^{2}+C_{1}\right)\left(\sigma_{x}^{2}+\sigma_{y}^{2}+C_{2}\right)}$$

where *x* and *y* represent the two images to be compared; $u_{x}$ and $u_{y}$ represent the mean of images *x* and *y*, respectively. $\mu_{x}^{2}$ and $\mu_{y}^{2}$ are the variance of images *x* and *y* and $\sigma_{xy}$ is the covariance of images *x* and *y*. $C_{1}$ and $C_{2}$ are constants. The range of SSIM values is [−1,1], and the closer the SSIM is to 1, the more similar the two images are. Taking the example of incomplete penetration MO image, Table 2 shows the evaluation results of three different filtering algorithms.

It can be seen from the table that the MSE value of Gaussian filtering is the smallest, the PSNR value is the largest, and the SSIM value is the closest to 1. Although its filtering time is slightly longer than that of median filtering, its noise-reduction performance is superior. Additionally, after multiple comparisons of different MO images using various filtering algorithms, Gaussian filtering consistently produced the best results. Therefore, for the purpose of filtering MO images, Gaussian filtering stands out as the optimal choice.

## 3. Detection and Classification by BP Neural Network and SVM

### 3.1. Feature Extraction Based on PCA

Multi-angle weld defects were formed on the weld seam using the experimental method described in Section 2.1, including pit, crack, lack of penetration, gas pore, and no defects. The excitation voltage of the alternating magnetic field was set to 120 V, and the lift height of the magnetic field generator was consistently maintained at 20 mm. The sampling frequency of the MO sensor was set to 75 Hz. MO images of natural weld defects under alternating magnetic field excitation, including three consecutive frames of dynamic MO images, are shown in Table 3. After image denoising, the collected MO images of weld defects have a size of 400 pixel × 400 pixel. Its pixels are too large, which may affect the efficiency of classification as input to the classification model. To reduce the amount of information and still retain key features, a nearest neighbor method can be used to downsample the Gaussian filtered MO images.

**Figure 5:**
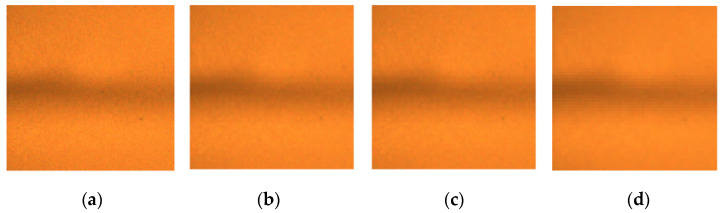
Downsampling of MO image. (**a**) 200 × 200; (**b**) 100 × 100; (**c**) 50 × 50; (**d**) 25 × 25.

The downsampled images still contain the main original information, serving as input for the subsequent principal component analysis to enhance feature extraction efficiency. Figure 5 shows the downsampling results of the MO image; it can be seen from Figure 5d that the image distortion is quite obvious. Therefore, a downsampling image with a size of 50 pixel × 50 pixel was ultimately selected.

PCA is a commonly used data dimensionality reduction technique that aims to transform original data into fewer features to extract the main information of the data [41,42]. The downsampled image is represented as a matrix ***X*** with dimensions 50 × 50. The data need to be centralized by subtracting the mean of each feature from its value, resulting in a standard normal distribution with a mean of 0 and a variance of 1 for each feature, which can be expressed as:

(8) $$Z_{ij}=\frac{X_{ij}-\mu_{j}}{\sigma_{j}}$$

where ***Z*** denotes the standardized matrix, $\mu_{j}$ represents the mean of the j column pixels, and $\sigma_{j}$ indicates the standard deviation of the j column pixels. The standardized MO image is represented as $Z=\left(z_{1},z_{2},z_{3},\cdots z_{50}\right)$, with $E\left(z_{j}\right)=0$, $D\left(z_{j}\right)=1$, j={1,2,3,⋯50}.

The principal components $X_{i}$ and the variance contribution rates $P_{i}$ of the column vector pixels in the MO image are given by:

(9) $$X_{i}=\sum_{k=1}^{50}\alpha_{ik}Z_{k}$$

(10) $$P_{i}=\frac{\lambda_{i}}{\sum_{k=1}^{50}\lambda_{k}}$$

Let P represent the sum of the variance contribution rates of the first *m* principal components, which can be expressed as:

(11) $$P=\frac{\Sigma_{i=1}^{m}\lambda_{i}}{\sum_{k=1}^{50}\lambda_{k}}$$

where $\alpha_{ik}Z_{k}$ denotes the product of the principal component’s feature vector and the standardized matrix, and *λ* refers to the eigenvalue of the correlation coefficient matrix of the downsampled MO images. P is set to 95%.

**Figure 6:**
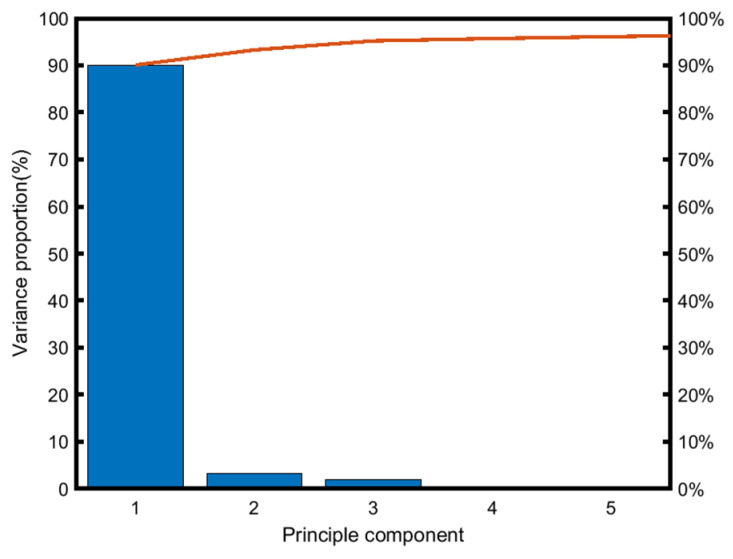
Principal components proportion of a downsampling MO image of weld defects.

After processing a downsampled MO image through image processing methods to extract the variance contribution of PCA column vectors as shown in Figure 6, it can be observed that the sum of the variance contribution rates of the preceding three principal components in the MO image of weld defects accounts for more than 95% of the information in the original image. Considering the varying contributions of principal components across different downsampled MO images, to adequately extract the original information of the image, ***m*** is set to 4.

After applying PCA, the image feature matrix is transformed to 50 × 4, converting the original feature matrix with a size of 200 × 1. The first 50 rows correspond to the first principal component of the MO image, followed by the subsequent 50 rows that correspond to the second principal component; then, another 50 rows correspond to the third principal component; finally, the last 50 rows correspond to the fourth principal component. The information of the image can be compressed into the four principal components, thus realizing the dimension reduction processing. A total of 1250 MO images of five types of natural weld defects were collected under the excitation of an alternating magnetic field. After Gaussian filtering and downsampling, the principal components of the column vectors of each defect MO image were reduced. The four-dimensional principal components of the column vectors were selected to be retained, forming an image input matrix of 200 × 1250, which was used as the input set for the BP neural network classification model and SVM classification model.

To expand the sample, the MO image is improved as follows:

(1) Resize: Resize the original image either horizontally or vertically.
(2) Rotation: Make the original image rotate clockwise or counterclockwise according to a certain angle.
(3) Flip: Flip the original images along the horizontal or vertical axes at their center coordinates.
(4) Brightness change: Change the brightness of the original image.
(5) Contrast adjustment: Change the intensity of the brightness difference in the original image.

**Figure 7:**
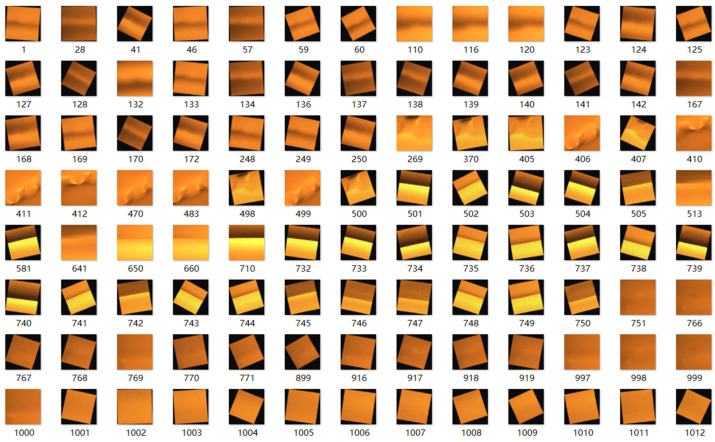
Part of weld defect’s MO images dataset.

The original number of defect’s MO images was expanded using the above method, and the expanded dataset is shown in Figure 7.

### 3.2. Classification by the PCA + BP Model

**Figure 8:**
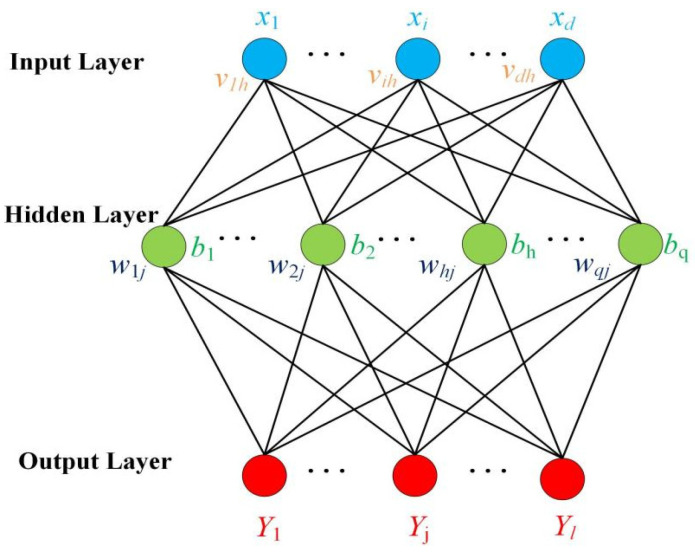
Topological structure of the BP neural network.

The principle of the BP neural network is to train the network through the backpropagation algorithm, enabling it to learn the mapping relationship between inputs and outputs [43,44]. PCA is a commonly used dimensionality reduction technique that helps improve the training efficiency and classification performance of subsequent BP by removing redundant features from the data. The use of PCA effectively reduces the number of input features, thereby reducing the complexity of the model, preventing overfitting, and improving the performance of the model on small sample datasets. As shown in Figure 8, the training set $D_{T}$ is defined as a dataset containing *m* samples, which can be expressed as:

(12) $$D_{T}=\left\{\left(x_{1},y_{1}\right),\left(x_{2},y_{2}\right),\ldots \left(x_{m},y_{m}\right)\right\}\;$$

where $\;x_{i}\in R^{d},y_{i}\in R^{l}$, meaning there are *d* input attributes and an output *l*- dimensional vector.

A multilayer forward feedback neural network defining an input neuron as *d*, hidden layer neurons as *q*, and output neurons as *l*. *v* and *w* are the weights of input layer and hidden layer and implied layer and output layer, respectively. For the hidden layer, the input and output of the *h*-th neuron can be represented by Equations (13) and (14), respectively:

(13) $$z_{h}=\sum_{i=1}^{d}v_{ih}x_{i}+b_{h}$$

(14) $$a_{h}=f_{1}\left(z_{h}\right)$$

where $x_{i}$ is the output value of the i neuron in the input layer, $b_{h}$ represents the deviation, and $f_{1}\left(z\right)$ is the activation function for the hidden layer. The hyperbolic tangent function is used as the activation function, which can be expressed as:

(15) $$f\left(x\right)=\frac{e^{x}-e^{-x}}{e^{x}+e^{-x}}$$

For the output layer, the input and output of the *j* neuron can be represented by Equation (16) and Equation (17), respectively.

(16) $$z_{j}=\sum_{h=1}^{q}w_{hj}a_{h}+c_{j}$$

(17) $$a_{j}=f_{2}\left(z_{j}\right)$$

where $c_{j}$ represents the deviation, and $f_{2}\left(z\right)$ denotes the activation function of the output layer, with the SoftMax function being utilized as the activation function in this study. During the training process, the weights and biases are updated through the BP algorithm to minimize the error. This includes the weights $w_{hj}$ and deviations $c_{j}$ from the hidden layer to the output layer, as well as the weights $v_{ih}$ and deviations $b_{h}$ from the input layer to the hidden layer.

The experiment used 1250 MO images of natural weld defects as classification samples, of which 1000 were used as the training set. In the training set, there were 200 samples each of crack, pit, lack of penetration, gas pore, and no defects. PCA is employed to extract the preceding four principal components, $P_{1}$, $P_{2}$, $P_{3}$, and $P_{4}$, from the vector pixel representations of the images. These components represent the primary features of the weld defects and form the input set for the classification model, denoted as $=\left\{X_{1j},X_{2j},\cdots X_{200j}\right\}$, where (j=1,2,3,⋯1250). The number of neurons in the hidden layer is set to q=20, and the output set is defined as $Y_{1j}=1,Y_{2j}=2,Y_{3j}=3,Y_{4j}=4,Y_{5j}=5$, with (j=1,2,3,⋯250) representing the output labels of the BP network model corresponding to crack, pit, lack of penetration, gas pore, and no defects, respectively. The learning rate is configured to 0.001, and the training employs the optimized Levenberg–Marquardt backpropagation algorithm.

**Figure 9:**
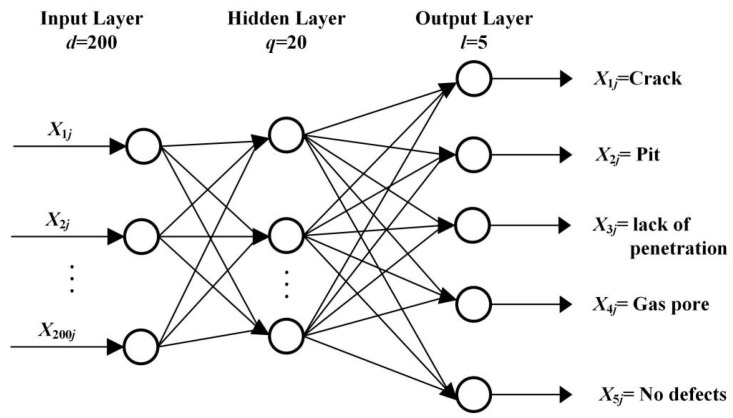
Classification model of the BP neural network for MO imaging of weld defects.

To prevent issues with the neural network during training, such as falling into local optima, the random initialization of weights and deviations are set. Figure 9 shows the classification model of the BP neural network for MO imaging of weld defects.

Table 4 shows the classification results of the BP neural network model for natural weld defects based on MO imaging. According to the table, the overall classification accuracy of the BP neural network model is 90.8%. Among the 250 images in the test set, 23 images were misclassified. Specifically, out of 50 MO images of cracks, 2 were classified as pit. Out of 50 MO images of pits, 2 were classified as cracks and 2 were classified as incomplete fusion. Out of 50 MO images of incomplete fusion, 5 were classified as gas pore. Out of 50 MO images of gas pore, 2 were classified as pit, 5 were classified as incomplete fusion, and 4 were classified as no defects. Out of 50 MO images of no defects, 1 was misclassified as gas pore. Thus, it is evident that the BP neural network classification model has poor performance in classifying pits and gas pores.

### 3.3. Classification by the PCA + SVM Model

SVM is a commonly used machine learning algorithm, mainly to find an optimal hyperplane given a dataset, so as to classify or regress the data, especially suitable for high-dimensional feature spaces [45,46]. The use of PCA provides SVM with a more effective feature set, thereby improving classification accuracy and helping SVM find the best decision boundary in high-dimensional space. The hyperplane in SVM can be represented using the linear Equation (18):

(18) $$\omega^{T}+b=0$$

where *ω* denotes the normal vector of the hyperplane, $\omega^{T}$ is the transpose of the vector *ω*, and *b* represents the bias term.

Let the sample set be $\{\left(x_{i},\;y_{i}\right),i=1,\;\ldots ,\;n$, where n represents the sample size, which in this study is n=1250, and $y_{i}$ is the label for the i sample $x_{i}$.

To solve the optimal hyperplane of the SVM, an optimization function is constructed to find the maximum value of the Lagrangian multiplier, which can be expressed as:

(19) $$max\left(\alpha \right)=\Sigma_{i=1}^{n}\alpha_{i}-\frac{1}{2}\sum_{\begin{array}{c} i=1\\ j=1 \end{array}}^{n}y_{i}y_{j}\alpha_{i}\alpha_{j}K\left(x_{i}\cdot x_{j}\right)$$

where $\alpha_{i}$ corresponds to the Lagrange multiplier for the i sample, and $K\left(x_{i},x_{j}\right)$ is the kernel function. The term $\frac{1}{2}\overset{n}{\underset{\begin{array}{c} i=1\\ j=1 \end{array}}{\sum }}y_{i}y_{j}\alpha_{i}\alpha_{j}K\left(x_{i}\cdot x_{j}\right)$ represents the similarity between samples, as computed by the kernel function.

The commonly used types of kernel functions currently include linear kernel function, polynomial kernel function, and radial basis function (RBF). The linear kernel function is applicable only to linearly separable datasets and is incapable of addressing nonlinear issues. The polynomial kernel function has multiple undetermined coefficients and requires parameter tuning through methods such as cross-validation, which is quite complex. Conversely, The RBF does not require parameter adjustment and is suitable for processing nonlinear data. Therefore, the RBF is used, which can be expressed as:

(20) $$K\left(x_{i},x_{j}\right)=e^{-\gamma |\left|x_{i}-x_{j}\right|{|}^{2}}$$

where $x_{i}$ and $x_{j}$ are the feature vectors of the data points, and *γ* is a parameter of the kernel function that governs the distribution of sample points after mapping to the feature space.

The discriminant function of the SVM classifier is obtained by solving it, which can be expressed as:

(21) $$f\left(x\right)=sign\left(\sum_{i=1}^{n}\alpha_{i}^{*}y_{i}K\left(x_{i}\cdot x_{j}\right)+b^{*}\right)$$

where $\alpha_{i}^{*}$ and $b^{*}$ are the optimal solutions for the Lagrange multipliers and bias terms obtained through grid search, respectively. The sign function is a squashing function that maps a real number to a binary output value of either 1 or −1, used for the final classification decision.

The experiment utilized 1250 MO images of weld defects as classification samples, of which 1000 were used as the training set. There were 200 samples each of crack, pit, lack of penetration, gas pore, and no defects in the training set. These images were subjected to noise reduction and downsampling to pixels. PCA was conducted to extract the preceding four principal components, $P_{1}$, $P_{2}$, $P_{3}$, and $P_{4}$, which represent the main characteristics of weld defects. This resulted in a total of 200×1250 inputs being classified using an SVM multi-classification model. The classification results of the model are shown in Table 5, with an overall classification accuracy of 91.6%.

For the crack images, five were misclassified as pores and two were misclassified as having no defects. For the pit image, one image was misclassified as a crack and three images were misclassified as lack of penetration. For the lack of penetration image, one image was misclassified as a crack, one image was misclassified as a pit, and one image was misclassified as having no defects. For gas pores, six were misclassified as cracks. For no defects, one image was identified as having a lack of penetration. This section will utilize the reduced dimensional feature vectors obtained through PCA as classification samples, establishing a classification model for weld defects based on the BP neural network and SVM. The experimental results showed that the overall recognition rate of the BP classification model was 90.8%, and the overall recognition rate of the SVM classification model was 91.6%. During the experiment, MO images were continuously acquired along the weld seam, sometimes only local characteristics of the weld defects were captured, which also led to some pores to be misclassified as cracks, there is an obvious misclassification between these two types of samples. Therefore, the classification accuracy of pores is relatively low in the BP and SVM models. Due to the significant differences between no defects and pit and other samples, the recognition rate in both BP and SVM models can reach over 92%.

## 4. Detection and Classification by CNN and ResNet50

### 4.1. The Architecture of CNN

**Figure 10:**
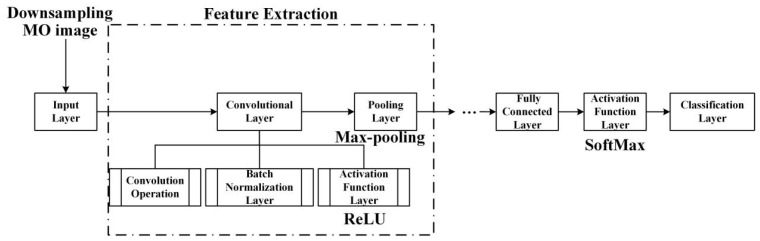
The structure flowchart of a CNN model.

CNN is a type of deep learning neural network that applies convolution operations on different parts of the input image to extract features and generate output, and then downsamples through pooling operations to reduce computational complexity and overfitting [47,48,49,50,51]. Then, the extracted features are transported to a fully connected layer, which classifies or regresses these features. The structural flowchart of a CNN model is shown in Figure 10. The primary components of a CNN consist of the input layers, convolutional layers, pooling layers, fully connected layers, and the output layers. CNN is a mainstream method in modern image processing and computer vision fields. Compared with traditional machine learning methods, CNN can automatically extract hierarchical features to improve efficiency.

Let an image of size $\left[W_{1}\times H_{1}\times C_{1}\right]$ be input, where $H_{1}$, $W_{1}$, and $C_{1}$ represent the height, width, and number of channels of the image, respectively, defining the image matrix as $\left[W_{1}\times H_{1}\times C_{1}\right]$. Consider a pixel located at (i,j), that feeds into the convolutional layer; this layer consists of multiple convolutional kernels. Each kernel performs convolution operations on the input image to extract various features. Let the size of each convolutional kernel be $K\times K\times C_{1}$, where $C_{1}=3$ means that the input has three channels. The convolution kernel has $C_{2}$, and the size of the output feature map is $W_{2}\times H_{2}\times C_{2}$. The convolution operation is expressed by Equation (22):

(22) $$z_{i,j,k}=\sum_{c=1}^{C_{1}}\;\sum_{p=1}^{K}\;\sum_{q=1}^{K}w_{\left(p,q,c,k\right)}x_{\left(i+p,j+q,c\right)}+b_{k}$$

where $z_{i,j,k}$ denotes the value at position (i,j) in the *k*-th channel of the output feature map, and k corresponds to the number of kernels and $k\leq C_{2}$. The term $w_{\left(p,q,c,k\right)}$ represents the weight for the *k*-th channel of the convolutional kernel at row p and column q of the *c*-th input channel, and $x_{\left(i+p,j+q,c\right)}$ indicates the pixel value at position (i+p,j+q) in the *c*-th channel of the input image. Consequently, for an input image with c channels and k convolutional kernels, there will be a total of c×k weight tensors utilized in the convolution operation. The term $b_{k}$ signifies the bias for the k channel.

The image after convolution operation enters a batch normalization (BN) layer, which standardizes the output of the convolution layer to improve training efficiency and model stability. The function of the activation layer is to introduce nonlinear factors and increase the expressive power of the neural network, which can be expressed as follows:

(23) $$a_{i,j,k}=f\left(y_{i,j,k}\right)$$

where f(⋅) is the activation function, and the commonly used activation functions include sigmoid, tanh, ReLU, etc. The ReLU is used as the activation function of the convolution layer, which is expressed as follows:

(24) $$f\left(x\right)=\{\begin{array}{c} 0,x<0\\ x,x\geq 0 \end{array}$$

After the convolution layer operation, it is followed directly by a pooling layer, which performs downsampling on the feature map, thereby reducing both the number of parameters and computational complexity while enhancing the model’s robustness. The pooling layer is typically categorized into max pooling and average pooling. This article adopts max pooling, and the pooling layer is represented as follows:

(25) $$P_{i,j,k}=\underset{p,q}{max}\left(a_{i\times s+p,j\times s+q,k}\right)\;$$

where $P_{i,j,k}$ represents the feature value at the i-th row and j-th column of the k-th channel output after pooling, with k as the size of the pooling window. The max function extracts the maximum value from a specified region. Variables p and q represent the row and column offsets of the pooling kernel in the input tensor, respectively. ***s*** is the step size, usually taken as 2.

After being processed by multiple convolutional and pooling layers, the output feature map will be flattened into a one-dimensional vector and entered into the fully connected layer for classification or regression tasks. The output formula of the fully connected layer is expressed as:

(26) F=WP+b 

where W represents the weight matrix with dimensions (N, M), and N is the number of neurons in the previous layer, and M denotes the number of neurons in the current layer. P corresponds to the vector obtained from the pooling layer after flattening into (N, 1), and b signifies the bias vector with a size of (M, 1), while F is the output vector. Following the fully connected layer, an activation function layer is applied, and this article uses the SoftMax activation function. Finally, the process culminates in the output layer for classification tasks. In convolutional neural networks, the cross-entropy loss function is used to measure the difference between the model’s predicted values and the true values. By minimizing the cross-entropy loss function, the prediction results of the model can be made closer to the true labels, thereby improving the accuracy of the model.

During training, a batch of data is used to calculate the loss function, and the gradient of the loss function for each trainable parameter is calculated for each image in this batch. This article uses the stochastic gradient descent (SGD) algorithm, which updates parameters using only one batch of data at a time [52]. For each trainable parameter, $w_{i}$, update its current value, $w_{i,t}$, as follows:

(27) $$w_{i,t+1}=w_{i,t}-\eta \frac{1}{m}\sum_{i=1}^{m}\frac{\partial L_{i}}{\partial w_{i}}$$

where η represents the learning rate, m is the number of images in the batch, and $L_{i}$ is the loss function for the i-th image, while $\frac{\partial L_{i}}{\partial w_{i}}$ is the gradient of the loss function of the i-th image with respect to $w_{i}$.

This paper adopts a learning rate scheduling method, that is, the learning rate is dynamically adjusted during the training to improve the performance of the model, which is expressed as follows:

(28) $$\eta =\eta_{0}d_{r}^{floor\left(\frac{g_{s}}{d_{s}}\right)}$$

where $\eta_{0}$ represents the initial learning rate, $d_{r}$ denotes the decay rate, which is constrained between 0 and 1. $g_{s}$ signifies the number of steps in the current iteration, and $d_{s}$ refers to the count of decay steps. The floor( ) function represents the process of rounding down. By managing the decay rate and the number of decay steps, it is feasible to reduce the learning rate, allowing for a higher initial learning rate to be established.

### 4.2. Parameter Evaluation of CNN Models

**Figure 11:**
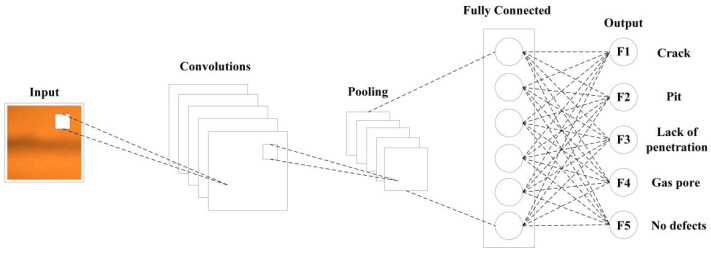
The CNN model for defect MO images.

To achieve optimal image classification performance, it is necessary to conduct experiments on the various structural parameters of the CNN prediction model in the classification of weld defect’s MO image, as well as to evaluate its accuracy and adaptability in classification tasks. The size of the convolution kernel directly influences the receptive field and the quality of the output features in deep convolutional neural networks. A larger convolution kernel size can obtain a smaller receptive field, and the output single feature can contain more information. However, a large size of the convolution kernel may lead to a surge in model computation, and the network is more difficult to train and optimize. Therefore, a network model with five convolutional layers, five pooling layers, and one fully connected layer was used in the experiment, as shown in Figure 11.

**Figure 12:**
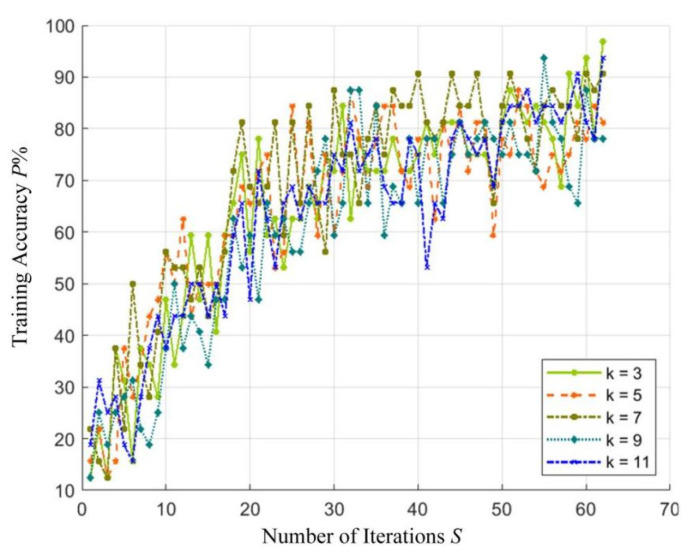
Training accuracy for different convolution kernel sizes.

**Figure 13:**
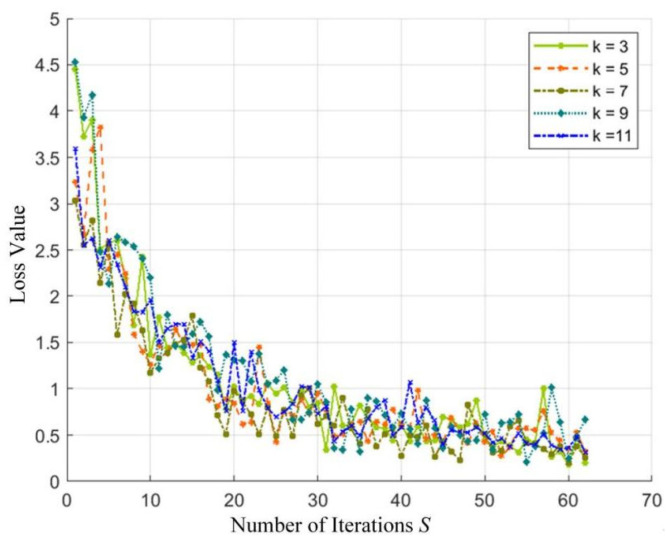
The training loss values associated with different convolution kernel sizes.

The effect of different structural parameters on the model is analyzed by comparing the effect of the convolution kernel size K×K in the first layer on the classification results. The odd-size convolution kernel has the advantages of facilitating the aggregation of image features, improving the robustness of the model, and easily expanding the network, so K=[3, 5, 7, 9, 11] is selected. In deep learning, accuracy and loss value are two commonly used evaluation indicators, which are used to measure the quality of training and model performance. The higher the accuracy and the smaller the loss value, the better the fit of the model. Figure 12 and Figure 13 show the training accuracy and loss values obtained under different convolution kernel sizes, respectively.

When the number of iterations, *S*, reaches 30, the accuracy of training reaches more than 70%, and the loss value also drops to around 1. Therefore, the intercept interval 30–62 is taken, and the standard deviation of the accuracy and loss value of training with different convolution kernel sizes is calculated, respectively, as shown in Table 6.

The standard deviation of accuracy for the 7 × 7 kernel size is the lowest, while the 5 × 5 kernel size shows the lowest standard deviation for loss values. A smaller standard deviation in accuracy and loss indicates a more stable optimization process, reflecting the stability and reliability of the model. Although the 11 × 11 convolution kernel has the highest accuracy, its standard deviation of accuracy is high, and the fitting effect between the recognition model and the training set is poor. Therefore, considering all factors, the optimal size for the convolution kernel in the first layer should be 7 × 7.

**Figure 14:**
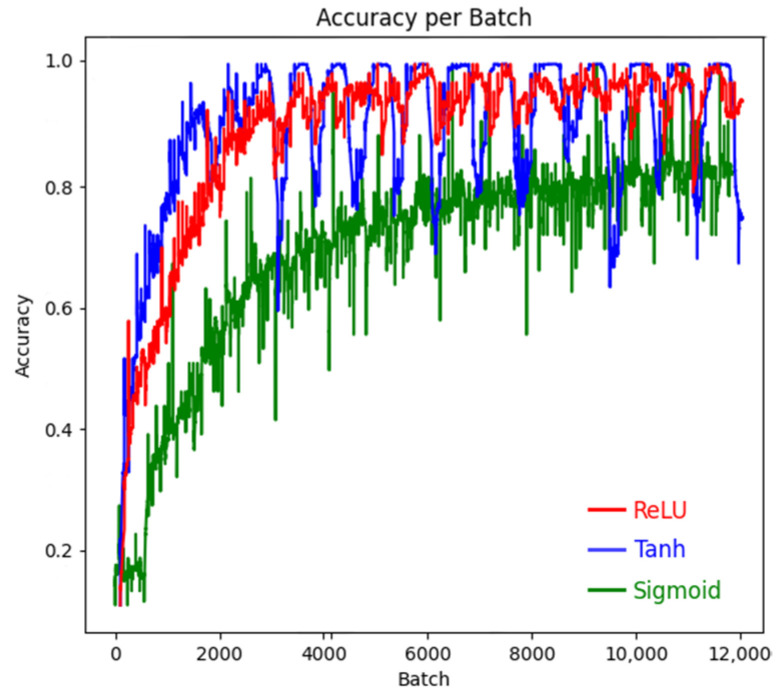
Training accuracies for three activation functions.

**Figure 15:**
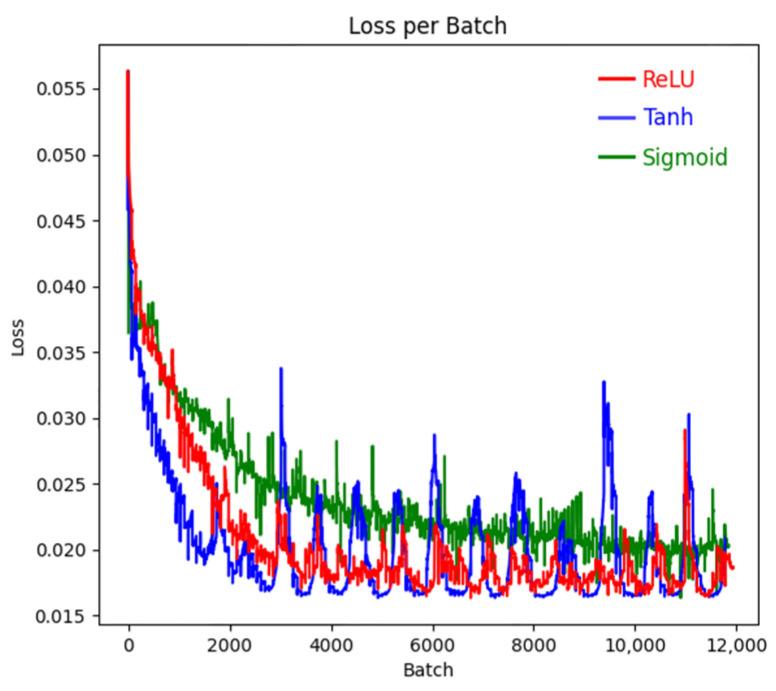
The loss values for the three activation functions.

In a neural network architecture, the excitation layer plays a key role in performing nonlinear transformation on the output of the convolutional layer. Without the excitation layer, no matter how complex the network architecture is, its final output will only be a linear superposition of the input, and it is difficult to capture the inherent law contained in the complex dataset. The essence of the excitation layer lies in the application of activation functions, such as Sigmoid function, Tanh function, and ReLU function, which are widely used activation functions. The slope of the activation function directly affects the speed of parameter iteration update, and the higher the slope, the faster the parameter update. The Sigmoid function and Tanh function have saturation zones, which can prevent parameter explosion during the training process. The Relu function has no saturation zone, and its advantage is that it can ensure the update iteration speed within the positive interval. Different activation functions have their own characteristics in various classification models, and the differences are significant. Therefore, based on the above three typical activation functions, the performance of them in the MO image classification model of weld defects is analyzed, and the corresponding training accuracy and loss values are shown in Figure 14 and Figure 15. As can be seen from the figures, the loss value and accuracy of Sigmoid are poor. Although Tanh can achieve faster convergence, it is less stable and will produce large fluctuations. Therefore, ReLU activation function is more suitable for this classification model.

### 4.3. Classification by the CNN Model

According to the analysis in Section 4.2, the size of the first convolution kernel is 7 × 7, and the weights and biases of the convolution and fully connected layers are initialized using a normal distribution with a mean of 0 and a variance of 1. The scaling factor of the batch normalization layer is initialized to 1, and the bias is initialized to 0. The retention probability of the dropout layer is initialized to 0.5. Due to the significant impact of training parameters on model prediction performance, the optimal training parameters for the model were determined through experiments. This article uses the Adam optimizer for model training. The Adam optimizer combines momentum and adaptive learning rates, allowing for rapid convergence and effectively reducing the oscillation of parameter updates. Its adaptive mechanism provides each parameter with a unique learning rate, which is especially advantageous when handling high-dimensional data and complex models. Additionally, the bias correction function of the Adam optimizer enhances the stability during the initial training phase. Therefore, the choice of the Adam optimizer ensures efficient and stable optimization of the model throughout the entire training process. The primary initial parameters of the model are shown in Table 7.

Table 8 shows the operational configuration parameters of the computer. The structure of the CNN model for classifying these MO images of weld defects is illustrated in Table 9.

**Figure 16:**
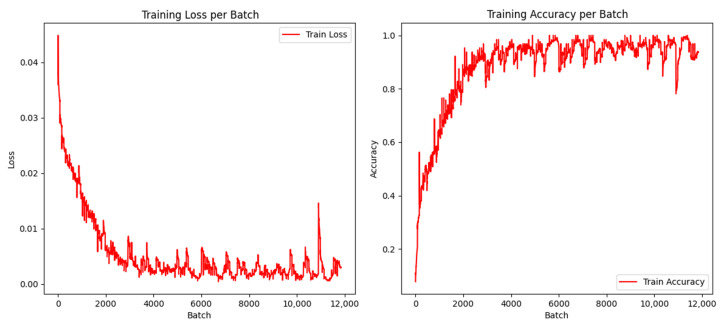
Training accuracy and loss value of CNN model.

**Figure 17:**
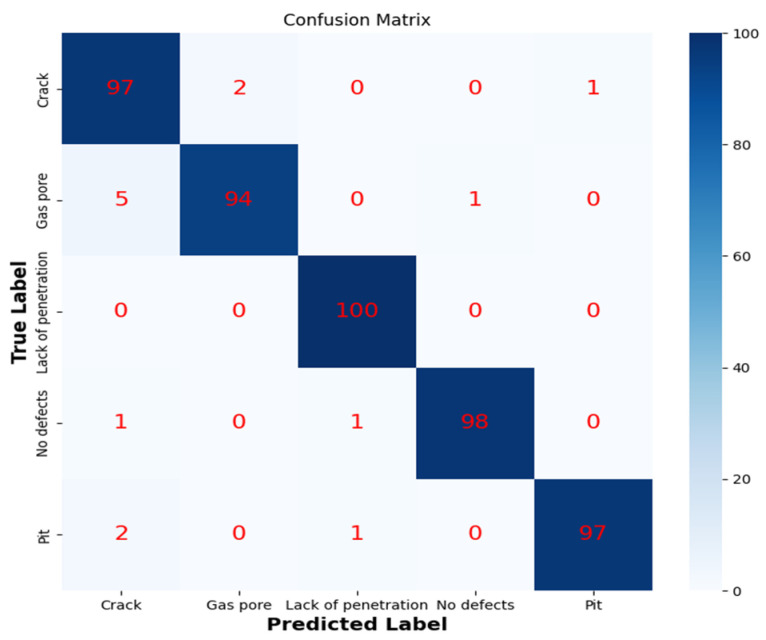
Confusion Matrix for CNN Classification.

The experiment used 5000 MO images of weld defects as classification samples, of which 4000 were used as training set. In the training set, there were 800 samples each of crack, pit, lack of penetration, gas pore, and no defects. After undergoing noise reduction and downsampling to 50 × 50 pixels, these images were used as the input for training a CNN model. The training accuracy and loss value of the CNN model are shown in Figure 16. In order to improve the generalization ability of the model, *K*-fold cross-validation method was adopted to divide the dataset. In this cross-validation method, the dataset was first divided into *K* mutually exclusive subsets of similar sizes, and *K* − 1 subsets were trained each time, and the remaining subsets were used for testing. The classification performance of the CNN on the training set is shown in Table 10. The CNN model has the best classification performance for lack of penetration, reaching 100%, with an overall classification accuracy of 97.2%. The classification results demonstrate that the model can effectively identify cracks, pits, incomplete fusion, gas pores, and no defects in the weldments. Therefore, it is feasible to use a multilayer CNN for the classification of weld defect MO images. Figure 17 shows the confusion matrix of the classification results. The classification effect of pores was the worst, and five pores were identified as cracks, indicating that the similarity between MO images of defects may cause confusion.

### 4.4. Classification by ResNet50 Model

**Figure 18:**
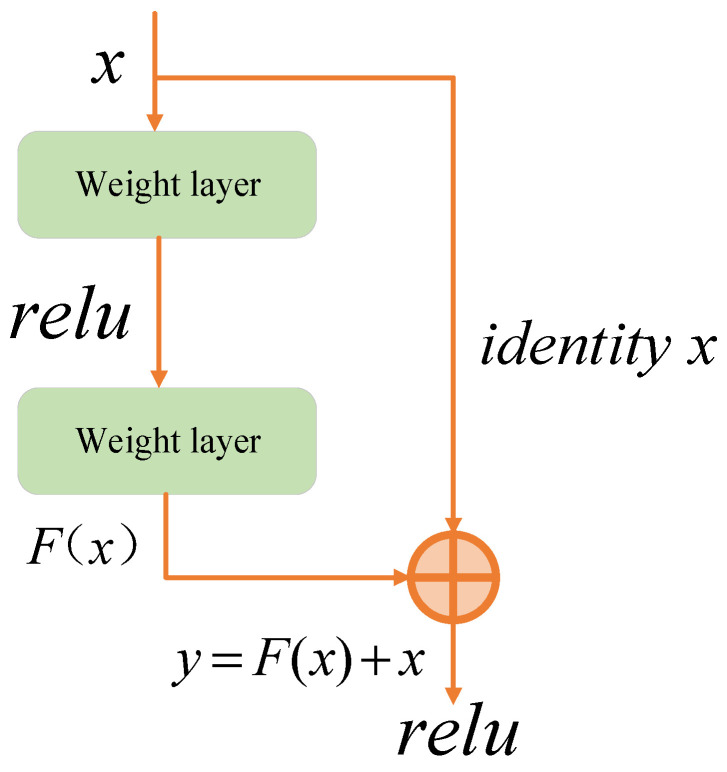
Simplified diagram of the residual module structure.

ResNet is an important improvement in comparison with traditional convolutional neural networks. Introducing the residual unit makes it easier for deep networks to learn the identity mapping, thus solving the degradation problem in the training process of deep neural networks. Compared with ordinary stacked deep neural networks, it is easier to optimize and obtain higher accuracy, thus showing superior performance in image classification. The residual module is the key of the ResNet network. Its structure is shown in Figure 18. After input x is obtained by convolution operation of the main line, the module performs addition operation with the eigenmatrix in the identity mapping. If the original base mapping that the network needs to fit is F(x), then there is Y=F(x)+x; that is, the residual module makes it so that the network does not need to fit the real base mapping, but only needs to fit the offset F(x) based on the original input identity mapping. So, there is no need to introduce additional parameters, reduce the computing burden of the network, or make the network performance at least not worse than the original. The ResNet network model has evolved into five structures, such as ResNet18, ResNet34, ResNet50, ResNet101, and ResNet152. With the increase in the number of network layers, the calculation accuracy of ResNet network is gradually improved. The number of computation loads and parameters also increases with the depth of the network. Considering accuracy, parameter counting, and calculation load, the ResNet50 network was used in this study [53]. During the training process, RTX2060 is used to classify and predict MO images of weld defects.

**Figure 19:**
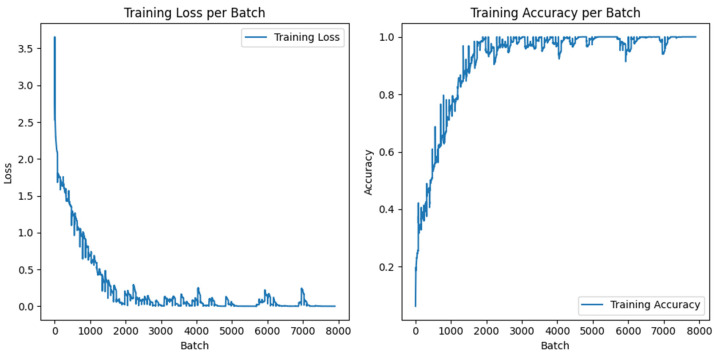
Training accuracy and loss value of ResNet50 model.

**Figure 20:**
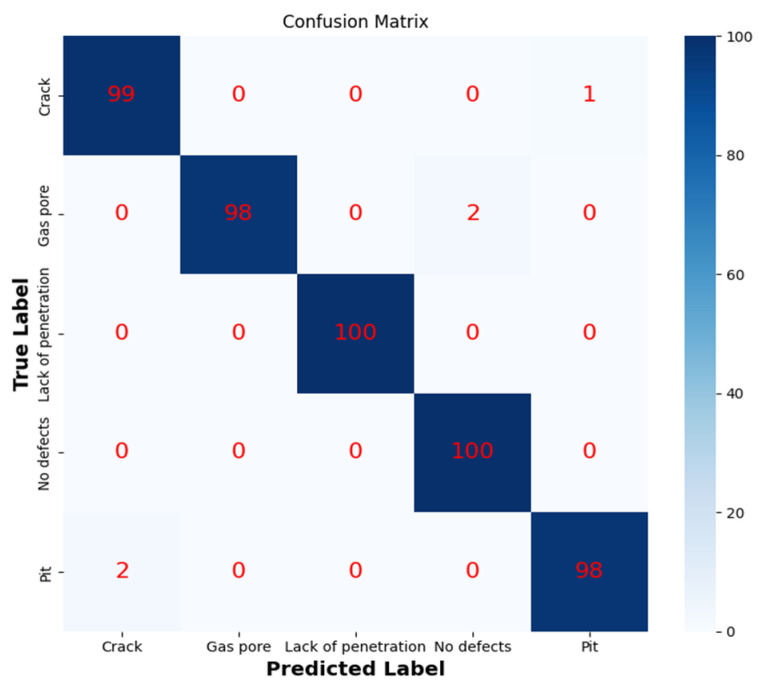
Confusion matrix for ResNet50 classification.

The experimental dataset is consistent with that in Section 4.3. A total of 4000 images were used as the training set, 500 as the validation set, and 500 as the test set. After noise reduction and downsampling to 50 × 50 pixels, these images were used as the input for training ResNet50. Model weights were updated using optimization techniques, and the model was gradually adapted to a specific classification task through repeated iterative training, and the performance of the model was evaluated on the test set. Figure 19 shows the loss value and accuracy of the ResNet50 training process. According to the data in Figure 19, the accuracy remains at a high level, which indicates that the ResNet50 network model has strong classification ability. The optimal recognition rate is obtained by constantly debugging the training times. The higher the training times, the better. The confusion matrix of the ResNet50 model is shown in Figure 20. The classification results of the ResNet50 model for MO images of weld defects are shown in Table 11. The overall classification performance is excellent, and the classification effect of incomplete fusion and gas pore is the best, both reaching 100%, and the overall classification accuracy is 99%.

### 4.5. Experimental Analysis

The classification methods proposed in this article include image downsampling, feature extraction, and PCA-BP, PCA-SVM, CNN, and ResNet50 classification models. The classification results for MO images of weld defects are shown in Table 4, Table 5, Table 10 and Table 11. The classification accuracy of the PCA-BP model is 90.8%. Since there are significant differences between cracks and no defects compared to other defects, the recognition rates can reach 96% and 98%, respectively. PCA-SVM shows a recognition rate of 94% for incomplete fusion and 98% for no defects. The recognition rates of cracks and gas pores are relatively low, at 86% and 88%, respectively. The classification accuracy of the PCA-SVM model can reach 91.6%, which is 0.8% higher than the PCA-BP model. However, the classification accuracy of gas pores in BP and SVM models is relatively low, which affects the overall classification accuracy. In Table 10, CNN achieves a recognition rate of 100% for MO images of incomplete fusion, demonstrating the model’s advantage in processing specific categories. However, the recognition rate for gas pores in MO images is the lowest at just 94%. Compared with the PCA-BP and PCA-SVM models, the classification accuracy of gas pores in the CNN model increased by 16% and 6%, respectively. The overall classification accuracy of the CNN model reaches 97.2%. Compared with PCA-BP and PCA-SVM models, the classification accuracy has been improved by 6.4% and 5.6%, respectively. Experimental results show that compared with the BP neural network model, the overall classification accuracy of the SVM classification model under alternating magnetic field reaches 91.6%, indicating that the SVM model has higher classification accuracy in small samples and high-dimensional pattern recognition. In large samples, the CNN and ResNet50 classification models are less prone to overfitting, and their classification accuracy has been greatly improved. These results highlight the effectiveness of different neural network models in classifying MO images of weld defects and emphasize the importance of deep learning methods in classification accuracy.

As shown in Figure 11, the ResNet50 model achieved classification accuracies of 99% for cracks and 98.0% for pores, surpassing the CNN model by 2% and 4%, respectively. The overall classification accuracy is 1.8% higher than the CNN model. This performance improvement may stem from the advantages of the ResNet50 model in feature extraction, particularly its effectiveness in capturing high-level features when processing complex images. In a deeper analysis of the experimental results, we observed that the classification accuracy for the four types of MO images (cracks, pits, lack of penetration, and no defects) exceeded 95% in both CNN and ResNet50 models, demonstrating good classification performance. The experimental results demonstrate the effectiveness of different neural network models in the classification of weld defect MO images, indicating that ResNet50 has certain advantages in this respect. The comparison of the four proposed algorithms is shown in Table 12. The parameters and floating-point operations per second (FLOPS) of the PCA-BP and PCA-SVM classification models are much lower than those of the CNN and ResNet50 models, which is due to their relatively simple network architectures. Despite the high computational complexity of CNN and ResNet50 deep learning models, they have performance advantages. Especially, the significant increase in accuracy is sufficient to compensate for the burden of computational complexity, which makes the ResNet50 model the best choice. These findings provide an important basis for further research on the identification and processing of MO images of weld defects. The forthcoming study will focus on improving the accuracy of target classification, especially in defect MO images with complex backgrounds, to further enhance the overall robustness of the system.

## 5. Conclusions and Outlook

The MO images of natural weld defects are obtained by the nondestructive testing system excited by an alternating magnetic field. The defect MO images featuring crack, pit, incomplete fusion, gas pore, and no defects have been acquired for weld defect diagnosis. After image enhancement and feature extraction of the collected defect MO images, PCA-BP model, PCA-SVM model, CNN model, and ResNet50 model are used to classify the MO images of natural weld defects.

Different image filtering techniques of weld defect images are compared, and the filtering effect of Gaussian filter, bilateral filter, and median filter is evaluated by mean square error. At the same time, the dimensionality-reduced feature vectors obtained by PCA are used as the input layers of BP neural network classification model and SVM classification model. The experimental results show that the overall recognition rate of the PCA-BP neural network classification model is 90.8%. The classification accuracy of the PCA-SVM model can reach 91.6%, which is 0.8% higher than the PCA-BP model. However, these two models have poor recognition performance for cracks and gas pores.

The CNN and ResNet50 classification models for MO imaging of natural weld defects are designed, and the forward propagation and iterative optimization process of the CNN model are studied. The experimental results show that the classification accuracy of the ResNet50 model is higher than that of the PCA-SVM model and CNN model, with an overall classification accuracy of 99%. Its total classification accuracy has increased by 7.4% and 1.8%, respectively. In particular, the classification accuracy of the gas pores is 10% and 4% higher than the PCA-SVM model and the CNN model, respectively, indicating that ResNet50 model can effectively improve the classification accuracy of MO images for natural welded defect. Therefore, the proposed method in this paper can provide high-precision diagnostic results for natural welded defects.

At present, the MO imaging detection technology of natural weld defects depends on the magnetic field excitation device, and different excitation modes have different imaging effects. Especially in the actual process of weld defect detection, there are many external factors that affect the effect of defect MO imaging, which require more theoretical research, simulation analysis, and practical experimental verification. Next, it is necessary to further develop a magnetic field excitation device and introduce frequency conversion function into the self-made excitation device to realize the MO imaging technology to detect weld defects at different frequencies, so as to meet the needs of automatic online detection. In future work, the author of this paper aims to investigate effective algorithms for automatically optimizing the parameters of the deep learning method and improved methods for reducing background noise to enhance accuracy. In addition, the method proposed in this article lays the foundation for extending the detection of welded defects using MO imaging technology under alternating magnetic field excitation to weld defect detection under rotating magnetic field excitation.

## Figures and Tables

**Table 1:** Main parameters of MO imaging sensor.

Light Source Wavelength	Sampling Frequency	Maximum Resolution	Pixel Equivalent	Magnetic Field Range
590 nm	[8, 100] fps	2592 × 1944 pixel^2^	102 pixel/mm	[−2, 2] kA/m

**Table 2:** Filtering results and indicators of different filtering algorithms for incomplete penetration.

Type	Filtering Result	MSE	PSNR (dB)	SSIM	Filtering Time (s)
Gaussian filtering	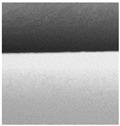	0.39	52.18	0.9956	0.1009
Bilateral filtering	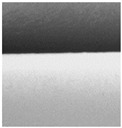	2.74	43.75	0.9656	0.8810
Median filtering	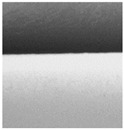	3.10	43.22	0.9684	0.0154

**Table 3:** MO images of five natural weld defects under alternating magnetic field excitation.

Defects	Frame 1	Frame 2	Frame 3
Pit	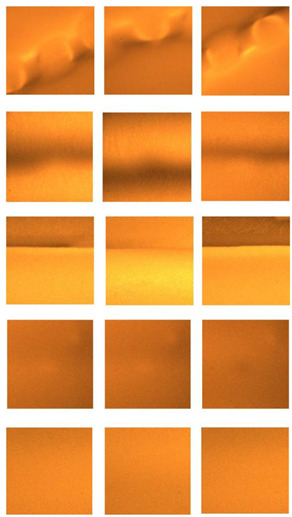		
Crack			
Lack of penetration			
Gas pore			
No defects			

**Table 4:** Classification results of the PCA-BP model for natural weld defects under alternating magnetic field excitation.

Defect Types	Number of Images	Train Samples	Test Samples	Recognition Result	Classification Accuracy/%
Crack	250	200	50	48	96
Pit	250	200	50	46	92
Lack of penetration	250	200	50	45	90
Gas pore	250	200	50	39	78
No defects	250	200	50	49	98
Total	1250	1000	250	227	90.8

**Table 5:** Classification results of the PCA-SVM model for natural weld defects.

Defect types	Number of Images	Train Samples	Test Samples	Recognition Result	Classification Accuracy/%
Crack	250	200	50	43	86
Pit	250	200	50	46	92
Lack of penetration	250	200	50	47	94
Gas pore	250	200	50	44	88
No defects	250	200	50	49	98
Total	1250	1000	250	229	91.6

**Table 6:** Training results of different convolution kernels.

Convolution Kernel Size	Accuracy Standard Deviation	Loss Value Standard Deviation	Training Set Accuracy %
3 × 3	0.073	0.207	93.2
5 × 5	0.073	0.160	96.4
7 × 7	0.067	0.175	97.2
9 × 9	0.082	0.216	92.8
11 × 11	0.084	0.170	97.6

**Table 7:** Initial parameters of the convolution neural network model.

Batch Size	Initial Learning Rate	Learning Rate Decay Factor	Number of Learning Rate Decay Steps	Number of Iterations
32	0.0002	0.1	1	62

**Table 8:** Parameters of computer operation configuration.

Deep Learning Framework	CPU	RAM	GPU	Operating Environment	Programming Language
Pytorch2.1.1	Intel(R)Core (TM)i7-10875 H	16 GB	NVIDIA GeForceRTX 2060	Anaconda 3	Python3.11.5

**Table 9:** Structural parameters of CNN model.

Layers	Types	Input Size	Filter Size	Number of Filters	Stride	Weights	Biases
I0	Input Layer	50 × 50 × 3	—	—	—	—	—
C1	Convolution Layer 1	50 × 50 × 3	7 × 7 × 3	32	1	7 × 7 × 3 × 32	1 × 1 × 32
P2	Pooling Layer 1	50 × 50 × 32	2 × 2	—	2	—	—
C3	Convolution Layer 2	25 × 25 × 32	3 × 3 × 32	64	1	3 × 3 × 32 × 64	1 × 1 × 64
P4	Pooling Layer 2	25 × 25 × 64	2 × 2	—	2	—	—
C5	Convolution Layer 3	12 × 12 × 64	3 × 3 × 64	128	1	3 × 3 × 64 × 128	1 × 1 × 128
P6	Pooling Layer 3	12 × 12 × 128	2 × 2	—	2	—	—
C7	Convolution Layer 4	6 × 6 × 128	3 × 3 × 128	256	1	3 × 3 × 128 × 256	1 × 1 × 256
P8	Pooling Layer 4	6 × 6 × 256	2 × 2	—	2	—	—
C9	Convolution Layer 5	3 × 3 × 256	3 × 3 × 256	512	1	3 × 3 × 256 × 512	1 × 1 × 512
P10	Pooling Layer 5	3 × 3 × 512	2 × 2	—	2	—	—
F11	Fully Connected Layer	1 × 1 × 512	—	—	—	5 × 512	5 × 1
S12	Classification Layer	1 × 1 × 5	—	—	—	—	—

**Table 10:** Classification results of the CNN model for MO images of weld defects.

Defect Types	Number of Images	Train Samples	Valid Samples	Test Samples	Recognition Result	Classification Accuracy/%
Crack	1000	800	100	100	97	97
Pit	1000	800	100	100	97	97
Lack of penetration	1000	800	100	100	100	100
Gas pore	1000	800	100	100	94	94
No defects	1000	800	100	100	98	98
Total	5000	4000	500	500	486	97.2

**Table 11:** Classification results of the ResNet50 model for MO images of weld defects.

Defect Types	Number of Images	Train Samples	Valid Samples	Test Samples	Recognition Result	Classification Accuracy/%
Crack	1000	800	100	100	99	99
Pit	1000	800	100	100	98	98
Lack of penetration	1000	800	100	100	100	100
Gas pore	1000	800	100	100	98	98
No defects	1000	800	100	100	100	100
Total	5000	4000	500	500	495	99

**Table 12:** Comparison of the four proposed algorithms.

Methods	Params	FLOPS	Classification Accuracy/%	Complexity
PCA-BP	6.12 × 10^3^	6.17 × 10^3^	90.8	Low
PCA-SVM	3.01 × 10^5^	1.5 × 10^7^	91.6	Intermediate
CNN	1.94 × 10^6^	3.76 × 10^9^	97.2	High
ResNet50	2.35 × 10^7^	1.34 × 10^10^	99	Highest

## Data Availability

The original contributions presented in the study are included in the article, further inquiries can be directed to the corresponding authors.
